# Occult very small lung carcinoma with a solitary brain metastasis that is clinically diagnosed as cavernous hemangioma: a case report

**DOI:** 10.4076/1757-1626-2-7475

**Published:** 2009-08-19

**Authors:** Tadashi Terada

**Affiliations:** Department of Pathology, Shizuoka City Shimizu HospitalMiyakami 1231 Shimizu-Ku, Shizuoka 424-8636Japan

## Abstract

The author reports herein a case of occult very small lung carcinoma with a solitary brain metastasis that is clinically diagnosed as cavernous hemangioma, with an emphasis on pathologic findings. A 48-year-old Japanese man was admitted to our hospital complaining of mild paresis of left leg. Brain CT and MRI showed a solitary tumor (2 cm) with features of cavernous hemangioma in the right temporal lobe. Tumorectomy was performed, and it was pathologically undifferentiated carcinoma. An immunohistochemical analysis reveled that the carcinoma cells were positive for four types of pancytokeratin, cytokeratin (CK) 5/6, CK7, CK18, CK19, p63, and Ki-67 (78%). They were negative for high molecular weight CK, CK14, CK20, TTF-1, PE-10, melanosome, S100 protein, EMA, vimentin, CD34, myoglobin, CEA, p53, desmin, α-smooth muscle actin, chromogranin, synaptophysin, CD56, neuron-specific enolase, CD68, KIT, and PDGFRA. The positive CK7 and negative CK20 suggested lung origin, and cytokeratin profiles and positive CK5/6 and p63 suggested a squamous differentiation. The pathological diagnosis was undifferentiated carcinoma with squamous differentiation probably of lung origin. Later, systemic CT, MRI and PET were performed, and they detected a small lung tumor (8 mm) in the right apex. The lung biopsy revealed an undifferentiated carcinoma with focal squamous differentiation; the immunohistochemical findings were the same as those of the brain tumor. These findings suggest that occult very small lung carcinoma can metastasize to brain and such a metastasis may mimic cavernous hemangioma radiologically. Pathologic observations using many antibodies are very useful to determine the origin and histological type in solitary brain nodule.

## Introduction

Brain metastasis of lung carcinoma is common. In the majority of such an event, lung tumor was first recognized, and brain metastasis develops later. Lung cancer with a fist manifestation of brain metastasis is relatively rare [1,2]. In addition, occult very tiny lung cancer <10 mm with a first manifestation of solitary brain metastasis is rare [1,2].

A solitary small brain mass lesion is difficult to diagnose correctly. The important points of differential diagnosis are the density of the lesion, contour of the lesion, and edema of surrounding tissue.

In general, determination of the origin of metastatic malignancies is difficult pathologically. Immunohistochemistry was used to determine this. However, because specific antibodies are a few (such as PSA for prostatic carcinoma), a panel of antibodies are used. Nevertheless, determination of the origin and histological type are frequently difficult.

The author herein reports a case of occult very small lung carcinoma presenting as a small solitary brain tumor that was clinically diagnosed as primary brain cavernous hemangioma. Pathological and immunohistochemical examinations revealed that the lesion was metastatic undifferentiated carcinoma with squamous differentiation, and could determine the origin and histological type of the brain tumor. Later, systemic CT, MRI and PET revealed a very small lung lesion, biopsy of which showed an undifferentiated lung carcinoma with squamous differentiation.

## Case presentation

**Figure 1.**
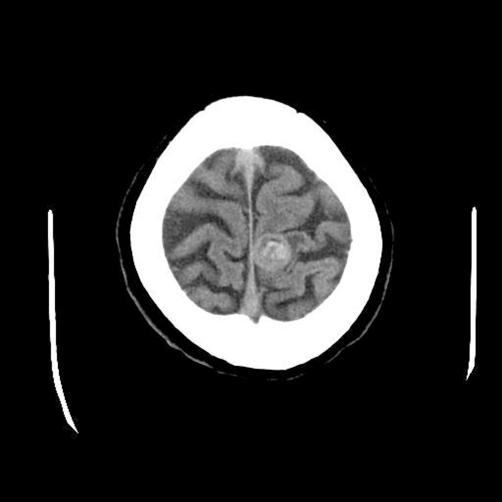
Brain CT. A solitary tumorous lesion (2 cm) is seen in the right temporal lobe. The tumor was solitary and well defined. No infiltrative growth was recognized. The density was that of blood. No edema was seen in the surrounding brain. The ridiologists’ diagnosis was cavernous hemanigioma.

**Figure 2.**
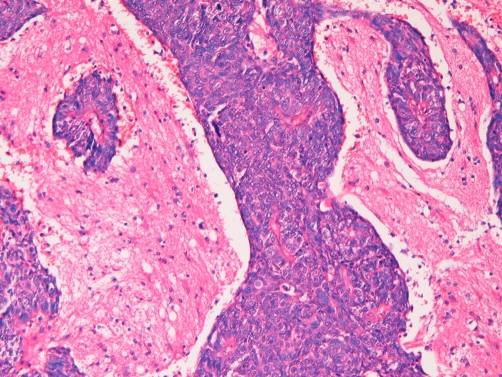
Histology of the brain tumor. Undifferentiated carcinomatous tissue is seen. The tumor cells were round and have hyperchromatic vesicular nuclei. Mitotic and apoptotic figures are scattered. No differentiation is seen. The pathological diagnosis was undifferentiated carcinoma. HE, ×100.

A 48-year-old Japanese man presented as mild paresis of left leg, and admitted to our hospital for scrutiny. Brain CT and MRI showed a solitary tumor (2 cm) with features of cavernous hemangioma in the right temporal lobe (Figure 1). The tumor was solitary and well defined. No infiltrative growth was recognized. The density was that of blood. No edema was seen in the surrounding brain. The radiological diagnosis was primary brain cavernous hemangioma. A tumorectomy was performed, and it revealed an undifferentiated carcinoma without differentiation on HE sections (Figure 2).

**Figure 3.**
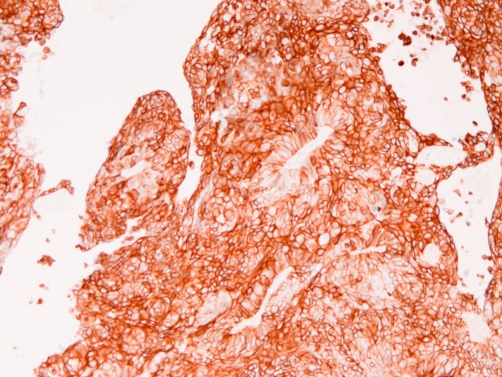
Cytokeratin 5/6 is strongly expressed in the cytoplasm of the brain tumor. ×100.

**Figure 4.**
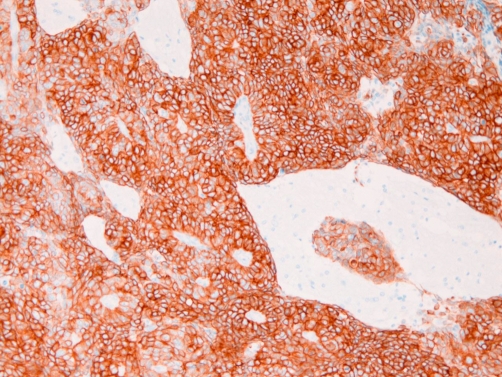
Cytokeratin 7 is strongly expressed in the cytoplasm of the brain tumor. ×100.

**Figure 5.**
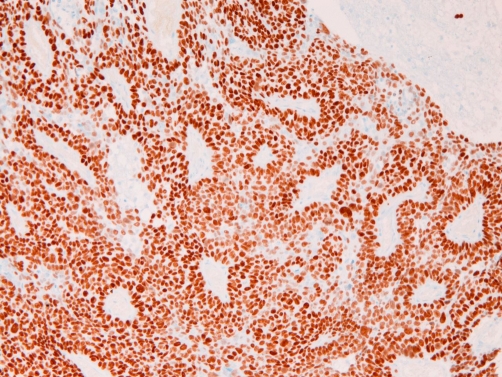
p63 is strongly and diffusely expressed in the nuclei of the brain tumor. ×100.

An immunohistochemical analysis was performed using Dako Envision method (Dako Corp. Glostrup, Denmark), as previously described [3-7]. The immnunohistochemical reagents and results are shown in (Table 1). The immunohistochemistry reveled that the carcinoma cells were positive for four types of pancytokeratins, cytokeratin (CK) 5/6 (Figure 3), CK7 (Figure 4), CK18, CK19, p63 (Figure 5), and Ki-67 (labeling = 78%). They were negative for high molecular weight CK, CK14, CK20, TTF-1, PE10. melanosome, S100 protein, EMA, vimentin, CD34, myoglobin, CEA, p53, desmin, α-smooth muscle actin, chromogranin, synaptophysin, CD56, neuron-specific enolase, CD68, KIT, and PDGFRA.

**Table 1.** Immunohistochemical reagents and results

Antigens	Antibodies (clone)	Sources	Results	
			Brain	Lung
Pancytokeratin	AE1/3	Dako Corp. Glostrup, Denmark	+++	+++
Pancytokeratin	polyclonal wide	Dako	+++	+++
Pancytokeratin	KL-1	Immunotech, Marseille, France	+++	+++
Pancytokeratin	CAM5.2	Bekton-Dicckinson, CA, USA	+++	+++
HMWCK	34βE12	Dako	−	−
CK5/6	D5/16	Dako	+++	+++
CK7	N1626	Dako	++	+++
CK14	LL002	Novocastra, Newcastle upon type, UK	−	−
CK 18	DC10	Dako	+	++
CK 19	RCK 108	Progen, Heidelberg, Germany	+	++
CK20	K20.8	Dako	−	−
TTF-1	8G7G3/1	Dako	−	−
Surfactant protein	PE-10	Dako	−	−
Melanosome	HMB45	Dako	−	−
EMA	E29	Dako	−	−
Vimentin	Vim 3B4	Dako	−	−
Myoglobin	polyclonal	Dako	−	−
CEA	polyclonal	Dako	−	−
Desmin	D33	Dako	−	−
S100 protein	polyclonal	Dako	−	−
CA19-9	NS19-9	TFB Lab, Tokyo, Japan	−	−
ASMA	1A4	Dako	−	−
CD34	NU-4A1	Nichirei, Tokyo, Japn	−	−
p53 protein	DO-7	Dako	−	−
p63	4A4	Dako	+++	+++
Ki-67	MIB-I	Dako	78%	62%
Chromogranin	DAK-A3	Dako	−	−
Synaptophysin	Polyconal	Dako	−	−
NSE	BBS/NC/VI-H14	Dako	−	−
CD56	UJ13A	Dako	−	−
CD68	KP-1	Dako	−	−
KIT	polyclonal	Dako	−	−
PDGFRA	polyclonal	Santa Cruz, CA, USA	−	−

+++, 67-100% positive; ++, 33-66%; +, 1-33% positive; −, negative; HMWCK, high molecular weight cytokerain; CK, cytokeratin; TTF-1, thyroid transcriptional factor-1; EMA, epithelial membrane antigen; CEA, carcinoembryonic antigen; ASMA, α-smooth muscle antigen; NSE, neuron-specific enolase; PDGFRA, platelet-derived growth factor receptor-α.

The positive CK7 and negative CK20 suggested a lung origin, and the cytokeratin profiles and positive CK5/6 and p63 suggested a squamous differentiation. The pathological diagnosis was undifferentiated carcinoma with squamous differentiation probably of lung origin.

**Figure 6.**
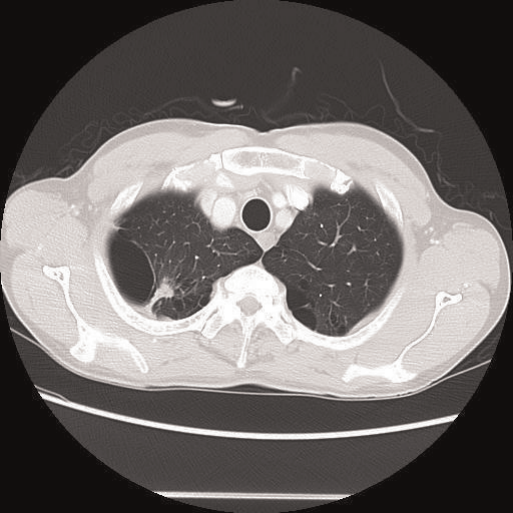
Chest CT. A very small tumor (8 mm) is seen in the right lung apex. The tumor shows irregular contours and invades the pleura.

**Figure 7.**
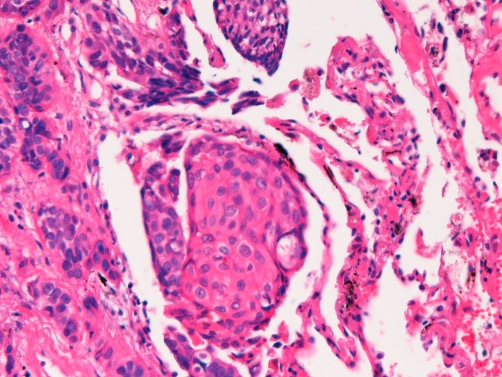
Histology of the lung tumor. Small foci of carcinoma cells are seen. Although most of them are undifferentiated, some show squamoid differentiation. It is interpreted as an undifferentiated carcinoma with focal squamous differentiation. ×100.

Later, systemic CT, MRI and PET were performed, and they detected a small lung tumor (8 mm) in the right apex (Figure 6). The lung biopsy revealed an undifferentiated carcinoma with focal squamous differentiation (Figure 7); the immunohistochemical findings were almost the same as those of the brain tumor (Table 1).

**Figure 8.**
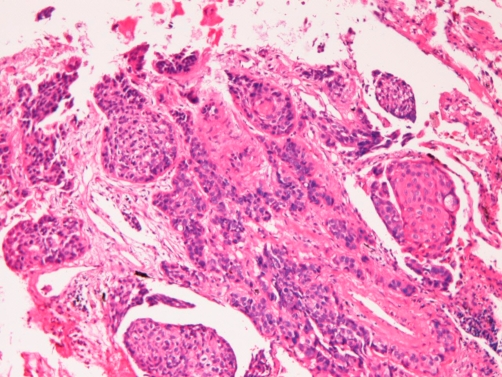
High power view of the Figure 7. The tumor shows ample acidophilic cytoplasm and abortive keratinization. The features are interpreted as squamous cell carcinoma. HE, ×200.

## Discussion

In the present case, the brain lesion was solitary and was clinically diagnosed as cavernous hemangioma based on the density, contour, and the absence of edema and invasive features which are findings of cavernous hemangioma [8]. These findings indicate that the metastatic lung carcinoma may mimic cavernous hemangioma of the brain radiologically. The author is experimental liver pathologist, and does not have enough knowledge of CT and MRI diagnosis. After the pathologic diagnosis of metastatic undifferentiated carcinoma, the author discussed radiologists the nature of brain shadow. They claimed that the lesion was solitary and well defined, and had the density of blood or hematoma No edema or invasive features were absent. Their retrospective diagnosis was also that of cavernous hemangioma. However, they regretted that metastatic carcinoma might show such a cavernous hemangioma-like shadow. The misdiagnosis of radiologists may be due to the difficulty in the interpretation of the shadow.

In the present study, the author examined 33 antibodies. The author myself performed these the large number of immunohistochemical staining not only for practical use but also for academic interest. In general, anti-pancytokeratins AE1/3 and CAM5.2 were adequate. The both antibodies detect wide ranges of cytokeratin. The author stained polyclonal and tibody and KL-1 for academic interest. High-molecular weigh cytokeratin (34βE12) and CK5/6 cover squamous epithelium. CK5/6 is used from distinction from mesothelioma. CK7/CK20 patterns were widely used as diagnostic pathology. The use of CK14 was of academic interest, but CK14 is useful in esophageal squamous cell carcinoma. The use of CK18 and CK19 was of academic interest, but these were used for distinction from pancreaticobiliary carcinoma. PE10 and TTF-1 were used as markers of adenocarcinoma of the lung. Melanosome and S100 protein were used from differentiation from epithelioid malignant melanoma. EMA and CKs were used to determine the epithelial nature of the tumor. The mesenchymal markers including vimentin, myoblobin, desmin, S100 protein, α-smooth muscle actin, CD34, and CD68 were used for differentiation from carcinomatoid sarcoma. P63 was used for squamous and transitional cell carcinoma. CEA was used for differentiation from poorly differentiated adenocarcinoma. P53 and Ki-67 was used for the malignant nature of the tumor and for the proliferative fraction. Chromogranin, synaptophysin, CD56, neuron-specific enolase were used to determine the neuroendocrine features of tumor cells. KIT and PDGFRA were used for neuroendocine carcinoma as well as some particular neoplasm. The author stained KIT and PDGFRA, because the author has investigated these molecules and mutational status of these genes.

The brain tumor was pathologically metastatic undifferentiated carcinoma on HE sections in the present case. Immunohistochemically, the tumor was positive for p63, various types of cytokeratin, CK 5/6, suggesting that the carcinoma had squamous differentiation [9-11]. Negative immunoreactions for TTF-1 and PE10 suggest that the tumor had no adenocarcinomatous differentiation [9-11]. The absence of neuroendocrine antigens (chromogranin, synaptophysin, CD56, neuron-specific enolase) suggests that the brain tumor was not neuroendocrine carcinoma such as large neuroendocrine carcinoma. Thus, the author could determine the histological type of the present brain tumor.

The majority of solitary metastasis of brain tumor was lung cancer, gastrointestinal cancers, and breast cancers [12]. In the present study, the CK7 and CK20 pattern suggested a lung cancer together with squamous nature of the tumor [13,14]. Considering the frequency of metastatic brain tumor and the present immunohistochemical study, the author could suggest that the tumor was lung undifferentiated adenocarcinoma with squamous differentiation. In fact, later imaging modalities detected a very small occult lung cancer (8 mm) in the right apex. The lung tumor was found to have the same histology and immunohistochemical findings as the brain tumor. Therefore, the present case was diagnosed as a occult lung carcinoma with a solitary brain metastasis.

In summary, the present case suggests that an extensive immunohistochemical investigation can determine histological type and origin in a solitary metastatic brain cancer. In addition, it was suggested that occult very small lung carcinoma can metastasize to brain and such a metastasis may mimic cavernous hemangioma radiologically. Pathologic observations using many antibodies are very useful to determine the origin and histological type in solitary brain nodule.
